# A Greek Case–Control Replication Study of *IKZF1* rs4132601 and *CDKN2A* rs3731217 in Childhood Acute Lymphoblastic Leukemia

**DOI:** 10.3390/genes17060682

**Published:** 2026-06-10

**Authors:** Ioannis Kyriakidis, Spyridoula D. Katsarou, Maria I. Zervou, Nikolaos Katzilakis, Maria Stratigaki, Iordanis Pelagiadis, Eftichia Stiakaki

**Affiliations:** 1 Department of Pediatric Hematology-Oncology and Autologous Hematopoietic Stem Cell Transplantation Unit, University Hospital of Heraklion and Laboratory of Blood Diseases and Childhood Cancer Biology, School of Medicine, University of Crete, 71003 Heraklion, Greece; kyriakidis@med.uoc.gr (I.K.); medp2012273@med.uoc.gr (S.D.K.); katzilaher@yahoo.gr (N.K.); mstratigaki@hotmail.com (M.S.); ipelagiadis@uoc.gr (I.P.); 2 MSc Program “Hematology-Oncology of Childhood and Adolescence”, School of Medicine, University of Crete, 71003 Heraklion, Greece; 3 Section of Molecular Pathology and Human Genetics, Department of Internal Medicine, School of Medicine, University of Crete, 71003 Heraklion, Greece; zervou@uoc.gr

**Keywords:** acute lymphoblastic leukemia, child, adolescent, *IKZF1*, rs4132601, *CDKN2A*, rs3731217, polymorphism, germline susceptibility, Greek population

## Abstract

Background/Objectives: Inherited variants in *IKZF1* and *CDKN2A/2B* are among the most consistently reported germline susceptibility markers for childhood acute lymphoblastic leukemia (ALL). Nonetheless, effect sizes differ across ancestry groups, age ranges, and immunophenotypic subtypes, making well-characterized population-specific replication studies valuable for refining ancestry-specific evidence. This study examined two sentinel variants with historical relevance, *IKZF1* rs4132601 and *CDKN2A* rs3731217, within a pediatric Greek cohort. Methods: A case–control study with retrospective case ascertainment and control recruitment through routine pediatric visits was conducted, comprising 50 children and adolescents with ALL and 91 healthy controls from Crete, Greece. Constitutional DNA was isolated from peripheral blood samples collected during remission in cases, while controls provided peripheral blood for targeted germline genotyping. Genotyping was performed using PCR-restriction fragment length polymorphism analysis. We evaluated Hardy–Weinberg equilibrium and genotype and allele distributions and analyzed the data using logistic regression models. Results: Control minor allele frequencies were broadly compatible with public European reference data. Neither *IKZF1* rs4132601 nor *CDKN2A* rs3731217 showed a significant association with susceptibility to childhood ALL under either genotype-based or additive models. Results remained consistent after excluding T-cell ALL cases. Exploratory genotype-phenotype analyses did not reveal robust associations with clinical or molecular features. Conclusions: In this first Greek pediatric assessment of *IKZF1* rs4132601 and *CDKN2A* rs3731217, no significant association with ALL susceptibility was observed. Within the constraints of limited statistical power, these negative findings provide population-specific evidence, refine regional allele-frequency and effect-size estimates, highlight the limitations of relying on single historical sentinel single-nucleotide polymorphisms (SNPs), and support future ancestry-informed and polygenic approaches in southeastern European cohorts.

## 1. Introduction

Childhood acute lymphoblastic leukemia (ALL) is the most prevalent pediatric neoplasia and results from a multistep process involving inherited predisposition, somatic genomic lesions, and developmental context. Although the majority of children with ALL do not possess highly penetrant germline cancer predisposition variants, genome-wide association studies (GWAS) have demonstrated that common inherited polymorphisms significantly contribute to ALL susceptibility. Multiple loci, including *ARID5B*, *IKZF1*, *CEBPE*, and *CDKN2A/2B*, have been consistently associated with B-cell precursor ALL (BCP-ALL), with some estimates indicating that known common variants account for a substantial yet incomplete portion of the inherited risk [1,2,3,4,5,6].

*IKZF1* encodes IKAROS, a zinc-finger transcription factor central to lymphoid development, chromatin remodeling, and early B-cell differentiation. The common variant rs4132601 at 7p12.2 was identified in early childhood ALL GWAS and has been associated with altered *IKZF1* expression in lymphoid cell models, supporting a biologically plausible link between inherited variation and leukemogenesis [1]. This germline signal is distinct from, but mechanistically relevant to, the well-established adverse prognostic role of somatic *IKZF1* deletions in B-ALL. *IKZF1* deletion and *IKZF1*^plus^ profiles are now incorporated into contemporary risk-stratification approaches across several protocols, particularly because these lesions frequently co-occur with high-risk genetic subtypes and are associated with inferior outcomes [7,8,9].

*CDKN2A* is located at 9p21.3, a genomic region known for its complex tumor suppressor functions. Through alternative transcripts, *CDKN2A* encodes the proteins p16INK4A and p14ARF, which are involved in cell cycle regulation and the p53 tumor suppressor pathway. The non-coding variant rs3731217 has been associated with an increased risk of childhood BCP-ALL in multiple large cohorts. Fine-mapping studies have identified rarer or more functionally relevant variants at the *CDKN2A* locus, including coding variants such as rs3731249 [2,3,5,6,10]. Functional data suggest that rs3731217 may affect *CDKN2A* transcript splicing or isoform expression, rather than overall gene expression [5].

Despite strong evidence from discovery, replication of *IKZF1* and *CDKN2A/2B* susceptibility variants has been inconsistent across studies. Differences in ancestry, age distribution, sample size, immunophenotypic composition, molecular subtype enrichment, DNA source, and genotyping strategy can all influence the observed associations. For example, *IKZF1* rs4132601 has shown clearer evidence in European, Hispanic, and some admixed cohorts than in several East Asian datasets, whereas *CDKN2A* rs3731217 has shown variable direction and significance across ancestry groups and age ranges [4,11,12,13,14,15,16,17,18]. These inconsistencies do not invalidate the original GWAS signals; rather, they emphasize that sentinel single-nucleotide polymorphisms (SNPs) may tag different causal haplotypes across populations and that modest genetic effects can be difficult to reproduce in small cohorts.

To our knowledge, no prior study has evaluated these two susceptibility loci in a pediatric Greek ALL cohort. Greece and the broader southeastern European region remain underrepresented in pediatric leukemia germline association research. Although existing large European GWAS and meta-analytic datasets provide the most precise estimates for these loci, they do not substitute for transparent regional replication in an underrepresented Greek pediatric cohort, particularly when local recruitment, DNA source, genotyping strategy, subtype composition, control allele frequencies, and Hardy–Weinberg equilibrium can be explicitly reported. Consequently, we conducted a Greek case–control replication study of *IKZF1* rs4132601 and *CDKN2A* rs3731217 in children and adolescents with ALL from Crete, using constitutional DNA collected during remission. The purpose was not to reaffirm known GWAS associations but to generate transparent, population-specific data, assess whether the expected effect direction was observed in this cohort, and interpret null results in the context of existing literature on ALL genetic susceptibility.

## 2. Materials and Methods

This case–control study included 141 participants from Crete, Greece. The case group was assembled retrospectively and comprised 50 children and adolescents with ALL treated at the Department of Pediatric Hematology-Oncology at the University Hospital of Heraklion. For cases, available clinical and laboratory data were retrospectively abstracted from institutional records and included age at diagnosis, sex, white blood cell (WBC) count at diagnosis, immunophenotype, major cytogenetic subgroup, relapse status, and vital status at last follow-up. High-risk classification, day 33 measurable residual disease (MRD), cytogenetic subgroup, and available *IKZF1* and *CDKN2A/B* deletion status were also abstracted from clinical records. MRD was assessed by flow cytometry, and *IKZF1*/*CDKN2A* deletion status by Multiplex Ligation-dependent Probe Amplification (MLPA). The control group was not identified through retrospective chart review. It comprised 91 unrelated healthy children from the same geographic area who were recruited through routine pediatric visits after guardian consent and provided peripheral blood for constitutional DNA extraction and targeted genotyping of the two study SNPs. Controls had no personal history of leukemia, hematologic malignancy, known cancer-predisposition syndrome, or chronic hematologic disorder. No broad leukemia diagnostic panel, MRD assessment, cytogenetic testing, or MLPA testing was performed in controls, because these investigations were clinically relevant only to patients with ALL. Thus, the retrospective component of the study refers to case ascertainment and abstraction of patient clinical/molecular data, whereas control recruitment and targeted germline genotyping were performed for the case–control comparison. To assess germline susceptibility, patient samples were obtained during remission to minimize the risk of contamination by disease-related somatic alterations.

Genomic DNA was extracted from peripheral blood using the PureLink Genomic DNA Mini Kit (Invitrogen, Thermo Fisher Scientific, Waltham, MA, USA). *IKZF1* rs4132601 and *CDKN2A* rs3731217 were genotyped by polymerase chain reaction-restriction fragment length polymorphism (PCR-RFLP) analysis using *MboI* and *MvaI*, respectively, according to manufacturer instructions and previously published protocols detailing primer sequences, amplicon sizes, restriction enzymes, and expected digestion fragments [19,20]. Genotyping was successful for all samples. Duplicate genotyping of 10% of samples showed complete concordance.

Hardy–Weinberg equilibrium (HWE) was assessed among controls using an exact test based on genotype counts. Control minor allele frequencies were also compared descriptively with public reference population frequencies to assess their plausibility in relation to broader European datasets. Genotype distributions were compared using either the Fisher–Freeman–Halton exact test or the Pearson chi-square test, as appropriate. Odds ratios (ORs) and 95% confidence intervals (CIs) were estimated using logistic regression under a log-additive model. Because of the limited number of rare homozygous genotypes, dominant, recessive, and overdominant models were interpreted descriptively rather than used to infer inheritance patterns. Associations between genotype and clinical or biological features were evaluated using the chi-square test, Fisher’s exact test, independent-samples *t*-test, Mann–Whitney U test, or Kruskal–Wallis test, depending on the data type and distribution. All statistical tests were two-sided, with significance set at *p* < 0.05. Analyses were conducted in IBM SPSS Statistics version 29.0.1.0 (IBM Corp., Armonk, NY, USA). Given the available sample size and observed control minor allele frequencies, the study was expected to detect only relatively large effects; therefore, null findings were interpreted cautiously and were not considered sufficient to exclude modest associations typical of common susceptibility variants [21]. Primary susceptibility analyses were unadjusted because the modest sample size and sparse genotype categories would make age- and sex-adjusted estimates unstable. The STROBE (Strengthening the Reporting of Observational Studies in Epidemiology) checklist is accessible in Table S1.

The study was conducted in accordance with the Declaration of Helsinki and approved by the Research Ethics Committee of the University of Crete (approval number 128/07.11.2023). Written informed consent was obtained from the legal guardians of all participants.

## 3. Results

### 3.1. Participant Characteristics

Participant characteristics are summarized in Table 1. The patient group included 50 children and adolescents with ALL, of whom 44 (88%) had B-lineage ALL and 6 (12%) had T-lineage ALL. The median age at diagnosis was 4.4 years, and the median WBC count at diagnosis was 12,900/μL. Relapse occurred in 5 patients (10%), and 45 patients (90%) were alive at last follow-up.

### 3.2. Association Between IKZF1 rs4132601, CDKN2A rs3731217 and ALL Susceptibility

Control minor allele frequencies were 22.5% for *IKZF1* rs4132601 and 12.6% for *CDKN2A* rs3731217, broadly compatible with public European reference frequencies. In exact HWE testing among controls, *CDKN2A* rs3731217 showed no relevant departure from equilibrium, whereas *IKZF1* rs4132601 showed genotype-level imbalance, characterized by fewer heterozygotes and more minor-allele homozygotes than expected. Accordingly, *IKZF1* genotype-specific results were interpreted with caution and were not considered definitive evidence for or against an association in this cohort. Genotype and allele distributions are shown in Table 2. No significant association with susceptibility to childhood ALL was observed for either variant. For *IKZF1* rs4132601, the log-additive OR per G allele was 0.845 (95% CI, 0.492–1.450; *p* = 0.541). For *CDKN2A* rs3731217, the log-additive OR per G allele was 0.755 (95% CI, 0.335–1.699; *p* = 0.497). Excluding the six T-ALL cases did not materially change the results. The rare *CDKN2A* rs3731217 GG genotype was observed in one patient and in none of the controls, precluding reliable inference under a recessive model.

### 3.3. Exploratory Clinicopathologic Analyses

Exploratory analyses did not identify robust associations between either SNP and available clinical or biological features, including sex, relapse, vital status, high-risk stratification, day 33 measurable residual disease, cytogenetic subgroup, and available *IKZF1* and *CDKN2A/B* deletion status. Two nominal findings were observed but deemed non-confirmatory: carriers of the *CDKN2A* rs3731217 TG genotype exhibited higher white blood cell counts at diagnosis compared to TT carriers (*p* = 0.023), and *IKZF1* rs4132601 TG carriers showed a tendency toward younger age at diagnosis relative to TT carriers (median comparison *p* = 0.06). These analyses were not corrected for multiple comparisons and were not used to support inferential conclusions. Given the limited sample size, sparse genotype representation, and multiple exploratory tests, these results should be interpreted only as hypothesis-generating observations.

## 4. Discussion

This pediatric case–control study conducted in Greece found no significant associations between *IKZF1* rs4132601 or *CDKN2A* rs3731217 and susceptibility to childhood ALL. This finding should be interpreted as a negative, underpowered regional replication rather than evidence of the absence of effect. Nevertheless, the study remains informative for three reasons. First, to our knowledge, this is the first investigation of these two loci within a Greek pediatric ALL cohort, a population not separately represented in most large datasets. Second, the study utilized remission-derived constitutional DNA, which is crucial in leukemia research because diagnostic blood or marrow samples may contain somatic leukemic mutations. Third, it provides transparent local data on control allele frequencies, HWE, subtype composition, and null effect estimates that can support future meta-analyses and help prevent selective publication of only positive candidate-variant results.

The absence of a statistically significant association should not be misconstrued as evidence that *IKZF1* or *CDKN2A/2B* biology is inconsequential to childhood ALL. Conversely, both genetic loci are integral to lymphoid leukemogenesis. IKZF1 plays a critical role in lymphoid differentiation, and somatic *IKZF1* deletions delineate biologically and prognostically high-risk B-cell ALL subsets. Deletions of *CDKN2A/B* are prevalent in both B-ALL and T-ALL and affect tumor-suppressor pathways that govern cell-cycle regulation and p53-mediated signaling. It is important to note that germline associations identified through sentinel SNPs and somatic alterations at the same loci address related but distinct biological questions [22,23,24].

Several explanations may account for our null findings. The most straightforward limitation is limited statistical power; therefore, this study cannot distinguish a true absence of effect in the local population from a false-negative result due to sample size and genotype sparsity. The expected effects of common susceptibility variants are typically modest, and our study lacked sufficient power to confidently exclude small or moderate associations. The control minor allele frequency for *IKZF1* rs4132601 was 22.5%, and for *CDKN2A* rs3731217 it was 12.6%, resulting in small genotype groups, especially for rare homozygotes. This was particularly relevant for *CDKN2A* rs3731217, where only one GG patient and no GG controls rendered recessive and codominant analyses unstable. A non-significant trend toward lower odds per minor allele was observed for both variants, but the confidence intervals were broad and consistent with either weak protective or weak risk effects.

Population-specific patterns of linkage disequilibrium present a plausible explanation. Sentinel SNPs identified in GWAS are not inherently causal; rather, they may serve as markers tagging causal variants that differ among ancestries. This disparity can weaken replication efforts when the study population diverges from the discovery cohort. The *CDKN2A* locus exemplifies this phenomenon, with rs3731217 historically serving as a significant marker. However, subsequent fine-mapping has revealed additional signals, including rare, high-impact coding variants such as rs3731249, which may account for the association at 9p21.3 in certain datasets [3]. With regard to *IKZF1*, rs4132601 remains a well-established GWAS marker at 7p12.2; however, recent fine-mapping studies have demonstrated that this locus may also harbor ancestry-specific susceptibility variants. Notably, a noncoding regulatory variant near *IKZF1* was recently identified as an independent risk signal for childhood ALL in Hispanic/Latino children but not in non-Hispanic White children, underscoring that associations within the *IKZF1* region may vary by ancestry and local linkage disequilibrium structure [25]. Accordingly, the absence of association at rs4132601 and rs3731217 does not rule out genetic susceptibility at these loci; rather, it indicates that these specific markers did not show measurable associations in the present cohort.

Age and immunophenotype may influence replication, with some susceptibility loci showing stronger effects in BCP-ALL than in T-ALL. Several studies have documented differences across age groups, including childhood, adolescent/young adult, and adult ALL [14,26]. Our cohort mainly consisted of B-lineage cases but also included a small number of T-ALL cases and adolescents. Excluding T-ALL did not alter the findings, though the cohort size was insufficient for robust subtype-specific analyses. Recognizing that modern ALL is a heterogeneous disease, subtypes such as *ETV6*::*RUNX1*, hyperdiploid ALL, Ph-positive ALL, Ph-like ALL, *TCF3*::*PBX1*, and *KMT2A*-rearranged ALL have distinct developmental origins and may exhibit different patterns of inherited susceptibility.

Our data further underscore the importance of cautious interpretation of genotype-phenotype associations in small candidate-SNP studies. The observed nominal association between *CDKN2A* rs3731217 heterozygosity and white blood cell count at diagnosis, along with the trend toward younger age among *IKZF1* rs4132601 heterozygotes, may be biologically significant but cannot be deemed conclusive without external validation. Similar prudence is warranted when evaluating the relationship between germline SNPs and somatic deletions. Previous research has proposed a potential interaction between inherited *IKZF1* variation and somatic *IKZF1* deletions in BCP-ALL [27], whereas data regarding *CDKN2A* variants and *CDKN2A/B* deletion status have been inconsistent [10,28]. In our cohort, no definitive association with available deletion status was identified.

Public GTEx Portal expression summaries offer an exploratory functional context for these variants. In whole blood, rs3731217 shows genotype-associated differences in *CDKN2A* expression in public GTEx data, whereas rs4132601 does not show a similarly clear whole-blood expression pattern for *IKZF1*. While these findings are biologically noteworthy, they should not be interpreted as direct mechanistic evidence for childhood ALL. Whole blood’s cellular heterogeneity and the absence of the relevant pre-leukemic progenitor context limit their applicability; additionally, previous functional investigations suggest that rs3731217 may influence transcript isoform usage or splicing rather than total gene expression alone [5,29]. Consequently, these public resource observations are best understood as supporting biological plausibility rather than establishing causality within this cohort. These public database observations were used only for exploratory biological contextualization.

The primary limitations of this study include its retrospective case ascertainment, modest sample size, limited statistical power, and the absence of genome-wide ancestry markers. While recruitment from a homogeneous population in Crete may reduce some heterogeneity, it does not fully eliminate population stratification. A specific limitation concerns the departure from HWE observed for *IKZF1* rs4132601 in controls. This imbalance could reflect random sampling variation in a modest control group, unrecognized local population structure or relatedness, or genotyping-related factors. The complete concordance observed in duplicate genotyping of 10% of samples reduces, but does not eliminate, the possibility of genotyping error, particularly because PCR-RFLP assays are less comprehensive than sequencing or array-based genotyping. Because HWE deviation in controls can bias genotype-specific association estimates and reduce confidence in a marker’s validity, the *IKZF1* rs4132601 findings were interpreted with caution and should not be considered definitive evidence for either a true null association or a protective/risk effect in the Greek population. Additionally, the study concentrated on two sentinel SNPs rather than employing a comprehensive polygenic risk score or a fine-mapped locus-wide panel. Furthermore, the analysis of clinical outcome correlations was exploratory, constrained by the number of relapses and the sizes of genotype-specific cell groups. Despite these limitations, reporting well-conducted negative replication data remains valuable. Such studies are crucial for refining GWAS effect-size estimates, mitigating publication bias, and avoiding overgeneralization of individual SNPs as universally applicable clinical markers across diverse populations.

Future investigations in Greece and southeastern Europe should extend beyond single-marker replication approaches. More informative study designs would include larger multicenter cohorts, collection of germline DNA during remission or from non-hematopoietic tissues, ancestry-informative genotyping, subtype-specific analyses, and development of polygenic risk models. Integration of inherited susceptibility with somatic genomic features, including *IKZF1* deletion, *IKZF1*^plus^ status, and *CDKN2A/B* deletion, may further elucidate whether constitutional genetic variation contributes to distinct biological pathways of leukemogenesis or influences treatment response. Pending the availability of such data, the present study serves as a transparent Greek reference dataset and supports a cautious interpretation of *IKZF1* rs4132601 and *CDKN2A* rs3731217 as standalone biomarkers.

## 5. Conclusions

To our knowledge, this is the first Greek pediatric study to examine associations between *IKZF1* rs4132601 and *CDKN2A* rs3731217 variants and the risk of childhood ALL. Neither variant showed a statistically significant association in this cohort. Because of the modest sample size and the *IKZF1* rs4132601 HWE imbalance in controls, the findings cannot distinguish a true null effect from a false-negative result for modest inherited effects. Even with this caution, the study provides useful population-specific negative replication data, documents local control allele distributions, and underscores the limitations of relying solely on single sentinel SNPs for risk prediction in understudied populations. To improve understanding of inherited ALL susceptibility in Greek and southeastern European pediatric cohorts, future research should include larger sample sizes, ancestry-informed analyses, subtype-specific investigations, and polygenic risk assessments.

## Figures and Tables

**Table 1:** Demographic and clinical characteristics of the study population.

Characteristic	ALL Patients (*n* = 50)	Controls (*n* = 91)
Age at diagnosis/enrollment, median (IQR; range), years	4.4 (5.9; 1.2–18.1)	6.2 (4.4; 1.5–17.5)
Sex, female/male	20 (40%)/30 (60%)	45 (49.5%)/46 (50.5%)
WBC at diagnosis, median (IQR; range), /μL	12,900 (11,850; 2300–498,000)	
Lineage, B/T	44 (88%)/6 (12%)	
B-ALL cytogenetics	Hyperdiploidy: 17 (38.6%); *ETV6*::*RUNX1*: 11 (25%); monosomies/near-diploid: 2 (4.5%); *BCR*::*ABL1*: 2 (4.5%); *TCF3*::*PBX1*: 2 (4.5%); *KMT2A*-r: 2 (4.5%); other: 8 (18.2%)	
Relapse	5 (10%)	
Alive at last follow-up	45 (90%)	

IQR, interquartile range; WBC, white blood cell count.

**Table 2:** Association analyses for *IKZF1* rs4132601 and *CDKN2A* rs3731217.

Variant/Model	Cases	Controls	Comparison *p*	OR (95% CI)	Model *p*
*IKZF1* rs4132601 TT	34 (68%)	60 (66%)	0.605	1.00	-
*IKZF1* rs4132601 TG	13 (26%)	21 (23%)		1.092 (0.478–2.438)	0.842
*IKZF1* rs4132601 GG	3 (6%)	10 (11%)		0.529 (0.113–1.870)	0.352
Allelic T/G	81 (81%)/ 19 (19%)	141 (77.5%)/ 41 (22.5%)	0.489		
Additive per G allele				0.845 (0.492–1.450)	0.541
*CDKN2A* rs3731217 TT	41 (82%)	68 (74.7%)	0.192	1.00	-
*CDKN2A* rs3731217 TG	8 (16%)	23 (25.3%)		0.577 (0.224–1.364)	0.226
*CDKN2A* rs3731217 GG	1 (2%)	0		Not estimable	-
Allelic T/G	90 (90%)/ 10 (10%)	159 (87.4%)/ 23 (12.6%)	0.510		
Additive per G allele				0.755 (0.335–1.699)	0.497

OR, odds ratio; CI, confidence interval. Genotype ORs use the common homozygous genotype as the reference group. The *CDKN2A* rs3731217 GG comparison was not estimable because no control carried this genotype. Control minor allele frequencies were consistent with public European reference data; however, *IKZF1* rs4132601 showed genotype-level imbalance among controls and was therefore interpreted with caution.

## Data Availability

The identified datasets used and analyzed during the current study are available from the corresponding author upon reasonable request, subject to institutional ethics approval and participant privacy restrictions.

## Supplementary Materials

The following supporting information can be downloaded at: https://www.mdpi.com/article/10.3390/genes17060682/s1, Table S1: STROBE checklist for case–control studies.

## Abbreviations

The following abbreviations are used in this manuscript:

ALL	Acute lymphoblastic leukemia
BCP	B-cell precursor
CI	Confidence interval
eQTL	Expression quantitative trait locus
GWAS	Genome-wide association studies
HWE	Hardy–Weinberg equilibrium
MLPA	Multiplex Ligation-dependent Probe Amplification
MRD	Measurable residual disease
OR	Odds ratio
PCR-RFLP	Polymerase chain reaction-restriction fragment length polymorphism
