# Supplementary material for: A Greek Case–Control Replication Study of IKZF1 rs4132601 and CDKN2A rs3731217 in Childhood Acute Lymphoblastic Leukemia
Source: Genes (Basel). 2026 Jun 10;17(6):682. doi: 10.3390/genes17060682 (PMC13300321; doi:10.3390/genes17060682)
Supplement: Supplementary file 1 [file genes-17-00682-s001.zip › genes-4359514-supplementary.pdf]

Table S1. STROBE checklist for case-control studies.

Section	Item No.	Recommendation	Reported on page(s)/location
Title and abstract	1a	Indicate the study design with a commonly used term in the title or the abstract	p. 1 (title); p. 2 (abstract)
Title and abstract	1b	Provide in the abstract an informative and balanced summary of what was done and what was found	p. 2
Introduction	2	Explain the scientific background and rationale for the investigation being reported	pp. 3-4
Introduction	3	State specific objectives, including any prespecified hypotheses	p. 4, final Introduction paragraph
Methods	4	Present key elements of study design early in the paper	p. 4, Methods - Study design and participants
Methods	5	Describe the setting, locations, and relevant dates, including periods of recruitment, exposure, follow-up, and data collection	pp. 4-5; Table 1, p. 5
Methods	6a	Give the eligibility criteria, and the sources and methods of case ascertainment and control selection. Give the rationale for the choice of cases and controls	pp. 4-5
Methods	6b	For matched studies, give matching criteria and the number of controls per case	Not applicable - non-matched case-control study
Methods	7	Clearly define all outcomes, exposures, predictors, potential confounders, and effect modifiers. Give diagnostic criteria, if applicable	pp. 4-6
Methods	8	For each variable of interest, give sources of data and details of methods of assessment. Describe comparability of assessment methods if there is more than one group	pp. 4-5
Methods	9	Describe any efforts to address potential sources of bias	pp. 4-6; limitations, p. 11
Methods	10	Explain how the study size was arrived at	p. 6
Methods	11	Explain how quantitative variables were handled in the analyses. If applicable, describe which groupings were chosen and why	p. 4; Table 1, p. 5
Methods	12a	Describe all statistical methods, including those used to control for confounding	pp. 5-6
Methods	12b	Describe any methods used to examine subgroups and interactions	p. 6
Methods	12c	Explain how missing data were addressed	p. 6
Methods	12d	If applicable, explain how matching of cases and controls was addressed	Not applicable - non-matched case-control study
Methods	12e	Describe any sensitivity analyses	pp. 6-7
Results	13a	Report numbers of individuals at each stage - e.g. numbers potentially eligible, examined for eligibility, confirmed eligible, included in the study, completing follow-up, and analysed	p. 5; Table 1, p. 5
Results	13b	Give reasons for non-participation at each stage	Not applicable/not available in this retrospective study; all included

Section	Item No.	Recommendation	Reported on page(s)/location
			participants were analyzed
Results	13c	Consider use of a flow diagram	Not used; compact case-control sample described in text and Table 1
Results	14a	Give characteristics of study participants and information on exposures and potential confounders	pp. 5-7; Tables 1-2
Results	14b	Indicate number of participants with missing data for each variable of interest	pp. 5-7; genotyping complete for all participants
Results	15	Report numbers in each exposure category, or summary measures of exposure	pp. 6-7; Table 2
Results	16a	Give unadjusted estimates and, if applicable, confounder-adjusted estimates and their precision. Make clear which confounders were adjusted for and why they were included	pp. 6-7; Table 2, p. 16. Unadjusted estimates reported; adjusted models were not performed because of modest sample size and sparse genotype categories
Results	16b	Report category boundaries when continuous variables were categorized	pp. 6-8; not applicable to primary SNP analyses
Results	16c	If relevant, consider translating estimates of relative risk into absolute risk for a meaningful time period	Not applicable - case-control susceptibility study; ORs reported
Results	17	Report other analyses done - e.g. analyses of subgroups and interactions, and sensitivity analyses	pp. 7-8
Discussion	18	Summarise key results with reference to study objectives	p. 7; Conclusions, pp. 10-11
Discussion	19	Discuss limitations of the study, taking into account sources of potential bias or imprecision. Discuss both direction and magnitude of any potential bias	p. 10
Discussion	20	Give a cautious overall interpretation of results considering objectives, limitations, multiplicity of analyses, results from similar studies, and other relevant evidence	pp. 8-11
Discussion	21	Discuss the generalisability (external validity) of the study results	p. 10
Other information	22	Give the source of funding and the role of the funders for the present study and, if applicable, for the original study on which the present article is based	p. 11

*Information is reported separately for cases and controls where relevant. Page numbers refer to the rendered submission-ready manuscript file.
